# Supplementary material for: Exploring heterogeneity in mosquito exposure and attraction and its implications for malaria transmission
Source: PLoS Comput Biol. 2026 Sep 8;22(9):e1014631. doi: 10.1371/journal.pcbi.1014631 (PMC13577544; doi:10.1371/journal.pcbi.1014631)
Supplement: Supplementary file 1 [file pcbi.1014631.s011.pdf]

Review of ”Exploring heterogeneity in mosquito exposure and attraction and its implications for malaria transmission”

by Kamber et al.

February 27, 2026

In this work, the authors use OpenMalaria, and individual-based stochastic modelling platform to assess the impact of heterogeneity in malaria transmission, particularly, heterogeneity in mosquito biting rates. Their work provides an interesting statistical framework for determining which probability distribution best characterize this heterogeneity. In general, the manuscript is well written (although with some typos) and the results are supported by relevant simulations. However, there are some comments that I believe can help to improve the manuscript.

Major comments

- Introduction, last paragraph of page 2. The authors mentioned the work of Dye and Hasibeder and also the work of Ross and Smith aiming to highlight the importance of heterogeneity in transmission models for vector-borne diseases. While these are indeed seminal contributions, the literature addressing heterogeneity in biting rates and transmission dynamics is considerably broader. The introduction would benefit from citing and briefly discussing additional relevant studies to better situate the present work within the existing body of research.
- Can the authors describe in more detail why they focus on the gamma and lognormal distributions to describe the heterogeneity between the rates at which individuals are bitten? are these the only valid distributions?
- The authors neglected seasonality in malaria transmission, however, it is well-know that seasonality plays a fundamental role in the transmission of most vector-borne diseases. Although the authors already mentioned this limitation in the Discussion, I believe more details need to be given specially in the light that the authors worked under equilibriums conditions. If I understand correctly, the authors assume the system reaches a stable steady state; however, in the presence of seasonality, such behavior may not occur, as the system could instead exhibit sustained oscillations. A more detailed discussion of how seasonality might affect the model outcomes and the validity of equilibrium assumptions would strengthen the manuscript.

Minor comments

- Page 2. The authors wrote ”basic reproductive rate” do they refer to the basic reproduction number R_0?
- Table 1 caption: Hyperparamters change to Hyperparameters
- Before eq. (4), the abbreviation CV is used but I believe it was not defined previously.
- Page 6, last sentence in the second paragraph the word negative is repeated twice.
- Page 6. The authors assume that individuals who are not infected before age 60 are excluded from malaria transmission. Could the authors clarify the motivation for this assumption and discuss its realism, given that individuals may live well beyond 60 years?

- Page7, marginal instead of maginal.

After addressing this comments, I recommend the publication of this manuscript.
