# Supplementary material for: Exploring heterogeneity in mosquito exposure and attraction and its implications for malaria transmission
Source: PLoS Comput Biol. 2026 Sep 8;22(9):e1014631. doi: 10.1371/journal.pcbi.1014631 (PMC13577544; doi:10.1371/journal.pcbi.1014631)
Supplement: Supplementary file 2 [file pcbi.1014631.s013.docx]

**Reviewer #1:** In this paper, Kamber et al. explore the implications of heterogenous exposure to mosquito-borne diseases on malaria transmission. They do so using a software called OpenMalaria, which is an agent-based model of malaria transmission that includes both vectors and humans. They find that heterogeneity leads to two effects, saturation effects and immunity development, that shift transmission patterns. Importantly, they estimate heterogeneity in malaria exposure using field data. They find that population-level incidence and prevalence decrease in the presence of greater heterogeneity in exposure.

The paper's strength is that it is fit to data, rather than these effects being observed in a theoretical simulation model.
Thank you for your positive feedback.

However, the usage of OpenMalaria is not sufficiently well-motivated, other than the fact that the authors developed the software. Explaining why it was best to use OpenMalaria rather than a much more simple model (which I think could have sufficed) would strengthen the manuscript.
We have added:
“OpenMalaria was selected because the epidemiological outcomes of interest (age-structured incidence and prevalence curves) are fundamentally shaped by acquired immunity, which accumulates in an age- and exposure-dependent manner over an individual's lifetime. Simple compartmental models lack the mechanistic representation of immunity necessary to reproduce these dynamics.”

Thus simple models could not meaningfully capture how heterogeneity in biting exposure interacts with immunity to alter disease burden across age groups. OpenMalaria's individual-based structure, with explicit simulation of immunity acquisition through repeated infection, is therefore essential for the analyses presented here. Furthermore, OpenMalaria has been extensively validated against field data across a wide range of transmission settings. This is particularly important when assessing whether heterogeneity improves model fit to field data.

Additionally, the manuscript does not refer to the literature on this topic nearly enough. For example, Smith et al 2004 ([https://journals.plos.org/plosbiology/article?id=10.1371/journal.pbio.0020368 [journals.plos.org]](https://urldefense.com/v3/__https:/journals.plos.org/plosbiology/article?id=10.1371*journal.pbio.0020368__;Lw!!PDiH4ENfjr2_Jw!E_EtOuGskwZG5U1ykbMkJzxNveeI8YrqTLP73zni8RT9vOWr_W8vxstVgihIHow5f4mQO__QEx6Irihw1-rYjyFBI1k$)) finds the same saturation effect the authors discover, albeit using a more theoretical model. In particular, the discussion is lacking citations and references to the literature. Relatedly, is this the first study that has fit data to a model incorporating heterogeneity? If not, previous studies should be cited - it's certainly implied that this is the case, given that there are no citations of additional studies.
We thank the reviewer for drawing our attention to Smith et al. (2004), which we had cited in an earlier version of the manuscript but removed during revision. We have now reinstated a citation to this paper in the Discussion, where we describe the saturation effect, adding the text: "This saturation effect, arising from the concave relationship between EIR and infection risk, was previously demonstrated theoretically (Smith et al. 2004), and empirically across African field sites (Smith et al. 2005), albeit without the immunity dynamics modelled here."

We note that Smith et al. (2004) addresses heterogeneity in EIR across spatial locations and time rather than individual-level biting heterogeneity within a population, and uses a simple model without immunity; nevertheless, the paper clearly demonstrates the same underlying saturation mechanism we observe, and we now explicitly acknowledge this.

In response to the broader point about engagement with the literature, we have thoroughly re-reviewed the relevant literature and made a number of additions throughout the manuscript. In the Introduction, we have expanded the discussion of prior work on heterogeneous exposure to include Woolhouse et al. 1997, Cooper et al. 2019, White et al. 2010, and Selvaraj et al. 2018, situating our work more fully within the existing body of research. In the Discussion, we have additionally cited Selvaraj at al. 2018 in the context of the interaction between seasonality and heterogeneity. Regarding whether this is the first study to fit data to a model incorporating heterogeneity: our primary novelty lies in providing a rigorous statistical framework for selecting an appropriate distribution for individual-level exposure heterogeneity and incorporating the resulting parameterisation into a fully calibrated, widely-used transmission model. We searched the literature for studies performing a comparable analysis and found only theoretical treatments, which are cited.

The emphasis of the gamma vs. log-normal distribution fitting the data better as a key result that appears even in the abstract is misguided in my opinion. This finding would be better stated by explaining the implications of what it means that a gamma distribution fit the data better. There should also be more of a focus on the practical implications of this study, rather than just a couple throw-away sentences at the end of the manuscript.
Added to abstract:
The gamma distribution better described the observed field data than the lognormal. This choice has a natural mathematical justification: when individual biting rates follow a gamma distribution and bites occur as a Poisson process, the resulting bite counts follow a negative binomial distribution, which is well-supported empirically for overdispersed count data of this kind. Furthermore, the gamma distribution's lighter tail produces more moderate saturation and immunity effects compared to lognormal-based models, yielding more realistic transmission dynamics.

Added to conclusion:
As transmission intensity declines, exposure becomes increasingly concentrated among a smaller proportion of the population (Cooper et al. 2019, Woolhouse et al. 1997). This suggests that standard interventions distributed uniformly across a community may be less efficient in elimination settings, where high-exposure individuals drive a disproportionate share of transmission (Cooper et al. 2019, White et al. 2010). Future work should explore methods for identifying and targeting these individuals to maximise the impact of limited resources

The incorporation of appropriate heterogeneity distributions into transmission models such as OpenMalaria enhances their suitability for guiding policy in low-transmission and elimination settings. Our results suggest that models assuming homogeneous exposure may systematically misrepresent the age distribution of disease burden and the relationship between EIR and epidemiological outcomes in these settings. Countries currently using OpenMalaria to inform elimination strategies should consider incorporating heterogeneity, particularly where observed age-incidence curves deviate from model predictions under homogeneous assumptions, as demonstrated here for sites in Mozambique and Cameroon.

Under heterogeneous transmission, a proportion of highly exposed individuals accumulate infections and develop immunity earlier in life, shifting the peak of age-incidence curves towards younger ages when comparing settings of equivalent prevalence. Conversely, less exposed individuals may fail to develop adequate immunity, potentially increasing clinical incidence in older age groups. Models that do not account for this heterogeneity may therefore misrepresent the age distribution of disease burden, with implications for the targeting of age-specific interventions such as seasonal malaria chemoprevention. Future work should explore how incorporating individual-level heterogeneity affects predictions of intervention impact in transmission models.

Hasibeder et al. 1988 examined how spatial clustering of vectors and hosts could further amplify transmission beyond individual-level heterogeneity, showing that strong associations between particular groups of mosquitoes and hosts could alter transmission dynamics. An extension of the present work would be the explicit inclusion of spatial heterogeneity in OpenMalaria through distinct mosquito populations. Similarly, the temporal variation in heterogeneity observed across transmission seasons, with greater heterogeneity during periods of lower transmission, suggests that static parameterisations of heterogeneity may be insufficient. Seasonal models that allow heterogeneity to vary with transmission intensity could provide more accurate representations of exposure dynamics, particularly in highly seasonal settings approaching elimination.

In summary, this study demonstrates that heterogeneous mosquito exposure is not merely a theoretical consideration but has substantive consequences for how malaria transmission is modelled and how control strategies are designed. The gamma distribution provides a statistically rigorous and practically meaningful characterisation of individual-level exposure heterogeneity, and its incorporation into OpenMalaria substantially improves model fit to field observations. As the malaria community works towards elimination, the concentration of transmission among a small proportion of highly exposed individuals means that models assuming homogeneous exposure risk misrepresenting both the epidemiological burden and the impact of interventions. Incorporating heterogeneity into transmission models such as OpenMalaria should therefore be considered a priority, particularly in low-transmission settings where its effects are most pronounced and the consequences of misspecification most consequential for policy.

In a similar vein, it would be helpful to include results that explicitly show the implications of not accounting for heterogeneity, for example simulating the adoption of an intervention and showing the differences in projected impact when accounting for vs. not accounting for heterogeneity. In this way, I think it would be clearer to modelers and decision-makers why more simplistic models may lead them astray even when it seems that the question at hand is "unrelated" to heterogeneity itself.
We thank the reviewer for this suggestion, which we agree would be a valuable contribution to the literature. However, we feel that a rigorous analysis of intervention impact under heterogeneous versus homogeneous exposure assumptions is beyond the scope of the present manuscript, which focuses on characterising the distribution of heterogeneity and its effects on fundamental epidemiological relationships at equilibrium. The impact of heterogeneity on intervention projections depends on multiple interacting factors including the mode of action of the intervention, its coverage, timing relative to transmission season, and the degree of targeting towards high-exposure individuals, and would warrant a dedicated study in its own right. We are currently preparing a companion paper that explicitly addresses how heterogeneity affects predictions of intervention impact in OpenMalaria, and this has been highlighted as a potential future direction in the discussion.

**Reviewer #2:**
In this work, the authors use OpenMalaria, and individual-based stochastic modelling platform to assess the impact of heterogeneity in malaria transmission, particularly, heterogeneity in mosquito biting rates. Their work provides an interesting statistical framework for determining which probability distribution best characterize this heterogeneity. In general, the manuscript is well written (although with some typos) and the results are supported by relevant simulations. However, there are some comments that I believe can help to improve the manuscript.

Major comments

• Introduction, last paragraph of page 2. The authors mentioned the work of Dye and Hasibeder and also the work of Ross and Smith aiming to highlight the importance of heterogeneity in transmission models for vector-borne diseases. While these are indeed seminal contributions, the literature addressing heterogeneity in biting rates and transmission dynamics is considerably broader. The introduction would benefit from citing and briefly discussing additional relevant studies to better situate the present work within the existing body of research.
Added:
Subsequent work has further characterised the implications of heterogeneous exposure across a range of contexts: Woolhouse et al. 1997 showed that heterogeneity in transmission has important consequences for the design of control programmes, whilst Cooper et al. 2019 demonstrated that Pareto-like rules govern malaria superspreading, with a small proportion of individuals driving a disproportionate share of transmission. The implications of heterogeneous exposure extend to intervention evaluation, with failure to account for heterogeneity shown to bias estimates of vaccine efficacy (White et al. 2010) and to affect predictions of intervention success more broadly Selvaraj et al. 2018).

• Can the authors describe in more detail why they focus on the gamma and lognormal distributions to describe the heterogeneity between the rates at which individuals are bitten? Are these the only valid distributions?
We thank the reviewer for this question. The justification for selecting the gamma distribution is grounded in its mathematical properties: when individual biting rates follow a gamma distribution and bites occur as a Poisson process, the marginal distribution of bite counts follows a negative binomial distribution, which is well-supported empirically for overdispersed count data of this kind. This connection is already noted in the Methods section. The lognormal was included as a comparator given its widespread use in modelling heterogeneous exposure in infectious disease settings. We acknowledge that other distributions could in principle be considered, and we discuss this in the Discussion, noting for example that triangular or nonparametric distributions leading to an 80/20 exposure pattern represent possible alternatives. However, the gamma and lognormal represent the most commonly applied and theoretically motivated choices for this type of data, and provide a meaningful and interpretable basis for comparison.

• The authors neglected seasonality in malaria transmission, however, it is well-know that seasonality plays a fundamental role in the transmission of most vector-borne diseases. Although the authors already mentioned this limitation in the Discussion, I believe more details need to be given specially in the light that the authors worked under equilibriums conditions. If I understand correctly, the authors assume the system reaches a stable steady state; however, in the presence of seasonality, such behaviour may not occur, as the system could instead exhibit sustained oscillations. A more detailed discussion of how seasonality might affect the model outcomes and the validity of equilibrium assumptions would strengthen the manuscript.

We thank the reviewer for this important technical point. We agree that the interaction between seasonality and equilibrium assumptions warrants more detailed discussion. Seasonality was excluded from the primary simulations in order to isolate the effects of individual-level heterogeneity and avoid confounding interactions between the two. We acknowledge the reviewer's point that in the presence of seasonality a static equilibrium does not exist; rather, the system settles onto a stable periodic orbit. Our 100-year burn-in period would be sufficient to reach this periodic steady state, so the validity of the equilibrium approach is maintained, though the nature of the equilibrium would change. We have expanded the Discussion to address this point directly, to explain the rationale for excluding seasonality more fully, and to highlight the temporal variation in heterogeneity we observed across transmission seasons as evidence of the complex interaction between the two factors that future work should address.

The discussion paragraph now reads:
Seasonality was excluded from the primary simulations in order to isolate the effects of individual-level heterogeneity and avoid confounding interactions between the two. In the presence of seasonal forcing, the system does not reach a static equilibrium but rather a stable periodic solution, returning to the same annual cycle each year. Our 100-year burn-in period is sufficient to ensure convergence to this periodic steady state. The interaction between seasonality and heterogeneity is complex: immunity acquisition depends on the timing and intensity of seasonal transmission peaks, and our analysis showed that the inverse correlation between EIR and heterogeneity may not only be applicable regionally, but also through seasonal variations in transmission. A larger CV was observed during the start and end of the season, when transmission levels were lower. This is consistent with previous modelling work demonstrating that heterogeneity in exposure plays a particularly important role in low-transmission settings, where it can substantially affect the success of interventions (Selvaraj et al. 2018). A full characterisation of the joint effects of seasonality and individual-level heterogeneity on transmission dynamics therefore represents an important direction for future work.

Minor comments

• Page 2. The authors wrote ”basic reproductive rate” do they refer to the basic reproduction number R0?
Changed

• Table 1 caption: Hyperparamters change to Hyperparameters
Changed

• Before eq. (4), the abbreviation CV is used but I believe it was not defined previously.
Definition added here and removed before next use.

• Page 6, last sentence in the second paragraph the word negative is repeated twice.
Deleted one

• Page 6. The authors assume that individuals who are not infected before age 60 are excluded from malaria transmission. Could the authors clarify the motivation for this assumption and discuss its realism, given that individuals may live well beyond 60 years?
We thank the reviewer for raising this point. The age of 60 was chosen as a practical threshold for defining the population at risk rather than as a biological claim about the susceptibility of older individuals. In high heterogeneity settings, a proportion of individuals receive very low exposure throughout their lives and may never experience an infection, effectively placing them outside the transmission cycle regardless of age. Tracking the age of first infection provides a natural way to quantify this effect, and age 60 represents a conservative upper bound for defining whether an individual has had meaningful opportunity to be exposed over a typical lifetime within the simulated population. We acknowledge that individuals may live beyond 60 and remain susceptible,

After: To assess the proportion of the population at risk, we track the age of each individual's first malaria infection. If individuals are not infected before the age of 60, we consider them to be excluded from malaria transmission

We have added:
this threshold was chosen as a conservative upper bound representing a typical lifetime of exposure opportunity within the simulated population, rather than as a biological claim about the susceptibility of older individuals.}

• Page7, marginal instead of maginal.
Fixed
