# Supplementary material for: Exploring heterogeneity in mosquito exposure and attraction and its implications for malaria transmission
Source: PLoS Comput Biol. 2026 Sep 8;22(9):e1014631. doi: 10.1371/journal.pcbi.1014631 (PMC13577544; doi:10.1371/journal.pcbi.1014631)
Supplement: Peer review history

# Peer review: Decision Letter 0

**DOI:** 10.1371/journal.pcbi.1014631.r001

**Authors:** Quirine ten Bosch, Benjamin Althouse

1 Mar 2026

PCOMPBIOL-D-25-02011

Exploring heterogeneity in mosquito exposure and attraction and its implications for malaria transmission

PLOS Computational Biology

Dear Dr. Fairbanks,

Thank you for submitting your manuscript to PLOS Computational Biology. After careful consideration, we feel that it has merit but does not fully meet PLOS Computational Biology's publication criteria as it currently stands. Therefore, we invite you to submit a revised version of the manuscript that addresses the points raised during the review process.

Please submit your revised manuscript by May 01 2026 11:59PM. If you will need more time than this to complete your revisions, please reply to this message or contact the journal office at ploscompbiol@plos.org. When you're ready to submit your revision, log on to https://www.editorialmanager.com/pcompbiol/ and select the 'Submissions Needing Revision' folder to locate your manuscript file.

Please include the following items when submitting your revised manuscript:

* A letter that responds to each point raised by the editor and reviewer(s). You should upload this letter as a separate file labeled 'Response to Reviewers'. This file does not need to include responses to formatting updates and technical items listed in the 'Journal Requirements' section below.

* A marked-up copy of your manuscript that highlights changes made to the original version. You should upload this as a separate file labeled 'Revised Manuscript with Track Changes'.

* An unmarked version of your revised paper without tracked changes. You should upload this as a separate file labeled 'Manuscript'.

If you would like to make changes to your financial disclosure, competing interests statement, or data availability statement, please make these updates within the submission form at the time of resubmission. Guidelines for resubmitting your figure files are available below the reviewer comments at the end of this letter

We look forward to receiving your revised manuscript.

Kind regards,

Quirine ten Bosch

Academic Editor

PLOS Computational Biology

Benjamin Althouse

Section Editor

PLOS Computational Biology

**Journal Requirements:**

1) Please ensure that the CRediT author contributions listed for every co-author are completed accurately and in full.

At this stage, the following Authors/Authors require contributions: Lars Kamber, Aurélien Cavelan, Melissa A Penny, Nakul Chitnis, and Emma Louise Fairbanks. Please ensure that the full contributions of each author are acknowledged in the "Add/Edit/Remove Authors" section of our submission form.

The list of CRediT author contributions may be found here: https://journals.plos.org/ploscompbiol/s/authorship#loc-author-contributions

2) We ask that a manuscript source file is provided at Revision. Please upload your manuscript file as a .doc, .docx, .rtf or .tex. If you are providing a .tex file, please upload it under the item type u2018LaTeX Source Fileu2019 and leave your .pdf version as the item type u2018Manuscriptu2019.

3) Please provide an Author Summary. This should appear in your manuscript between the Abstract (if applicable) and the Introduction, and should be 150-200 words long. The aim should be to make your findings accessible to a wide audience that includes both scientists and non-scientists. Sample summaries can be found on our website under Submission Guidelines:

https://journals.plos.org/ploscompbiol/s/submission-guidelines#loc-parts-of-a-submission

4) Please upload all main figures as separate Figure files in .tif or .eps format. For more information about how to convert and format your figure files please see our guidelines:

https://journals.plos.org/ploscompbiol/s/figures

5) We have noticed that you have uploaded Supporting Information files, but you have not included a list of legends. Please add a full list of legends for your Supporting Information files after the references list.

Note: If the reviewer comments include a recommendation to cite specific previously published works, please review and evaluate these publications to determine whether they are relevant and should be cited. There is no requirement to cite these works unless the editor has indicated otherwise.

**Reviewers' comments:**

Reviewer's Responses to Questions

**Comments to the Authors:**

**Please note that one review is uploaded as an attachment.**

Reviewer #1: In this paper, Kamber et al. explore the implications of heterogenous exposure to mosquito-borne diseases on malaria transmission. They do so using a software called OpenMalaria, which is an agent-based model of malaria transmission that includes both vectors and humans. They find that heterogeneity leads to two effects, saturation effects and immunity development, that shift transmission patterns. Importantly, they estimate heterogeneity in malaria exposure using field data. They find that population-level incidence and prevalence decrease in the presence of greater heterogeneity in exposure.

The paper's strength is that it is fit to data, rather than these effects being observed in a theoretical simulation model. However, the usage of OpenMalaria is not sufficiently well-motivated, other than the fact that the authors developed the software. Explaining why it was best to use OpenMalaria rather than a much more simple model (which I think could have sufficed) would strengthen the manuscript. Additionally, the manuscript does not refer to the literature on this topic nearly enough. For example, Smith et al 2004 (https://journals.plos.org/plosbiology/article?id=10.1371/journal.pbio.0020368) finds the same saturation effect the authors discover, albeit using a more theoretical model. In particular, the discussion is lacking citations and references to the literature. Relatedly, is this the first study that has fit data to a model incorporating heterogeneity? If not, previous studies should be cited - it's certainly implied that this is the case, given that there are no citations of additional studies.

The emphasis of the gamma vs. log-normal distribution fitting the data better as a key result that appears even in the abstract is misguided in my opinion. This finding would be better stated by explaining the implications of what it means that a gamma distribution fit the data better. There should also be more of a focus on the practical implications of this study, rather than just a couple throw-away sentences at the end of the manuscript. In a similar vein, it would be helpful to include results that explicitly show the implications of not accounting for heterogeneity, for example simulating the adoption of an intervention and showing the differences in projected impact when accounting for vs. not accounting for heterogeneity. In this way, I think it would be clearer to modelers and decision-makers why more simplistic models may lead them astray even when it seems that the question at hand is "unrelated" to heterogeneity itself.

Reviewer #2: Review comments are uploaded as an attachment

**********

**Have the authors made all data and (if applicable) computational code underlying the findings in their manuscript fully available?**

The PLOS Data policy requires authors to make all data and code underlying the findings described in their manuscript fully available without restriction, with rare exception (please refer to the Data Availability Statement in the manuscript PDF file). The data and code should be provided as part of the manuscript or its supporting information, or deposited to a public repository. For example, in addition to summary statistics, the data points behind means, medians and variance measures should be available. If there are restrictions on publicly sharing data or code —e.g. participant privacy or use of data from a third party—those must be specified.

Reviewer #1: None

Reviewer #2: Yes

**********

PLOS authors have the option to publish the peer review history of their article (what does this mean?). If published, this will include your full peer review and any attached files.

If you choose “no”, your identity will remain anonymous but your review may still be made public.

**Do you want your identity to be public for this peer review?**  For information about this choice, including consent withdrawal, please see our Privacy Policy.

Reviewer #1: No

Reviewer #2: No

[NOTE: If reviewer comments were submitted as an attachment file, they will be attached to this email and accessible via the submission site. Please log into your account, locate the manuscript record, and check for the action link "View Attachments". If this link does not appear, there are no attachment files.]

**Figure resubmission:**

After uploading your figures to PLOS’s NAAS tool - https://ngplosjournals.pagemajik.ai/artanalysis, NAAS will process the files provided and display the results in the "Uploaded Files" section of the page as the processing is complete. If the uploaded figures meet our requirements (or NAAS is able to fix the files to meet our requirements), the figure will be marked as "fixed" above. If NAAS is unable to fix the files, a red "failed" label will appear above. When NAAS has confirmed that the figure files meet our requirements, please download the file via the download option, and include these NAAS processed figure files when submitting your revised manuscript.

**Reproducibility:**

To enhance the reproducibility of your results, we recommend that authors of applicable studies deposit laboratory protocols in protocols.io, where a protocol can be assigned its own identifier (DOI) such that it can be cited independently in the future. Additionally, PLOS ONE offers an option to publish peer-reviewed clinical study protocols. Read more information on sharing protocols at https://plos.org/protocols?utm_medium=editorial-email&utm_source=authorletters&utm_campaign=protocols

Attachment Submitted filename: review.pdf

# Author comment: Author response to Decision Letter 1

**DOI:** 10.1371/journal.pcbi.1014631.r002

5 May 2026

Attachment Submitted filename: HeteroReviewComments.docx

# Peer review: Decision Letter 1

**DOI:** 10.1371/journal.pcbi.1014631.r003

**Authors:** Quirine ten Bosch, Benjamin Althouse

26 Jul 2026

Dear Dr Fairbanks,

We are pleased to inform you that your manuscript 'Exploring heterogeneity in mosquito exposure and attraction and its implications for malaria transmission' has been provisionally accepted for publication in PLOS Computational Biology.

Before your manuscript can be formally accepted you will need to complete some formatting changes, which you will receive in a follow up email. A member of our team will be in touch with a set of requests.

Please note that your manuscript will not be scheduled for publication until you have made the required changes, so a swift response is appreciated.

IMPORTANT: The editorial review process is now complete. PLOS will only permit corrections to spelling, formatting or significant scientific errors from this point onwards. Requests for major changes, or any which affect the scientific understanding of your work, will cause delays to the publication date of your manuscript.

Should you, your institution's press office or the journal office choose to press release your paper, you will automatically be opted out of early publication. We ask that you notify us now if you or your institution is planning to press release the article. All press must be co-ordinated with PLOS.

Thank you again for supporting Open Access publishing; we are looking forward to publishing your work in PLOS Computational Biology.

Best regards,

Quirine ten Bosch

Academic Editor

PLOS Computational Biology

Benjamin Althouse

Section Editor

PLOS Computational Biology

***********************************************************

The reviewers were satisfied with the responses and adaptations made to the manuscript. We are happy to say that this manuscript is deemed suitable for publication. We do want to ask you to consider the final notes by reviewer #2 (dr. Saldana) on the code availability and reproducibility.

Reviewer's Responses to Questions

**Comments to the Authors:**

**Please note here if the review is uploaded as an attachment.**

Reviewer #2: The authors have addressed correctly all my comments, so I agree with the publication of this work. Nevertheless, regarding code availability, although the authors shared the Github repository for the OpenMalaria software, at least for me, it' not clear that such repository includes the specific model developed for this study. In case not (and maybe even if yes), the authors must provide another repository dedicated to this work to aid reproducibility of these results. Furthermore, I believe a code availability statement should be added to the main manuscript where the link to the repository is clearly given.

**********

**Have the authors made all data and (if applicable) computational code underlying the findings in their manuscript fully available?**

The PLOS Data policy requires authors to make all data and code underlying the findings described in their manuscript fully available without restriction, with rare exception (please refer to the Data Availability Statement in the manuscript PDF file). The data and code should be provided as part of the manuscript or its supporting information, or deposited to a public repository. For example, in addition to summary statistics, the data points behind means, medians and variance measures should be available. If there are restrictions on publicly sharing data or code —e.g. participant privacy or use of data from a third party—those must be specified.

Reviewer #2: **No:** Regarding code availability, although the authors shared the Github repository for the OpenMalaria software, at least for me, it' not clear that such repository includes the specific model developed for this study. In case not (and maybe even if yes), the authors must provide another repository dedicated to this work to aid reproducibility of these results. Furthermore, I believe a code availability statement should be added to the main manuscript where the link to the repository is clearly given.

**********

PLOS authors have the option to publish the peer review history of their article (what does this mean?). If published, this will include your full peer review and any attached files.

If you choose “no”, your identity will remain anonymous but your review may still be made public.

**Do you want your identity to be public for this peer review?**  For information about this choice, including consent withdrawal, please see our Privacy Policy.

Reviewer #2: **Yes:** Fernando Saldaña

# Editor report: Acceptance letter

**DOI:** 10.1371/journal.pcbi.1014631.r004

**Authors:** Quirine ten Bosch, Benjamin Althouse

PCOMPBIOL-D-25-02011R1

Exploring heterogeneity in mosquito exposure and attraction and its implications for malaria transmission

Dear Dr Fairbanks,

I am pleased to inform you that your manuscript has been formally accepted for publication in PLOS Computational Biology. Your manuscript is now with our production department and you will be notified of the publication date in due course.

The corresponding author will soon be receiving a typeset proof for review, to ensure errors have not been introduced during production. Please review the PDF proof of your manuscript carefully, as this is the last chance to correct any errors. Please note that major changes, or those which affect the scientific understanding of the work, will likely cause delays to the publication date of your manuscript.

Soon after your final files are uploaded, unless you have opted out, the early version of your manuscript will be published online. The date of the early version will be your article's publication date. The final article will be published to the same URL, and all versions of the paper will be accessible to readers.

For Research, Software, and Methods articles, you will receive an invoice from PLOS for your publication fee after your manuscript has reached the completed accept phase. If you receive an email requesting payment before acceptance or for any other service, this may be a phishing scheme. Learn how to identify phishing emails and protect your accounts at https://explore.plos.org/phishing.

Thank you again for supporting PLOS Computational Biology and open-access publishing. We are looking forward to publishing your work!

With kind regards,

Sharmila Kamatchi

PLOS Computational Biology | Carlyle House, Carlyle Road, Cambridge CB4 3DN | United Kingdom ploscompbiol@plos.org | Phone +44 (0) 1223-442824 | ploscompbiol.org | @PLOSCompBiol
